# Serum 14-3-3η level is associated with severity and clinical outcomes of rheumatoid arthritis, and its pretreatment level is predictive of DAS28 remission with tocilizumab

**DOI:** 10.1186/s13075-015-0799-7

**Published:** 2015-10-09

**Authors:** Shintaro Hirata, Anthony Marotta, Yuan Gui, Kentaro Hanami, Yoshiya Tanaka

**Affiliations:** First Department of Internal Medicine, School of Medicine, University of Occupational and Environmental Health, 1-1 Iseigaoka, Yahatanishi, Kitakyushu 807-8555 Japan; Augurex Life Sciences Corporation, Vancouver, BC Canada

## Abstract

**Introduction:**

Treat-to-target strategies to achieve low disease activity or clinical remission are key in the treatment of rheumatoid arthritis (RA). 14-3-3η is a joint-derived biomarker that is expressed at significantly higher levels in patients with RA than in healthy subjects, other autoimmune diseases, or viral and bacterial arthritides. In this study, we sought to investigate the utility of pretreatment levels of 14-3-3η and serial measurement of 14-3-3η to inform therapeutic outcomes.

**Methods:**

Serum 14-3-3η levels were measured in 149 Japanese patients with RA before the initiation of therapy and at 1-year follow-up. Patients were treated with either methotrexate (MTX), adalimumab (ADA), tocilizumab (TCZ), or tofacitinib (TOF). 14-3-3η positivity was defined as ≥0.19 ng/ml and at two times and four times this cutoff. In contingency analysis, we determined the association of 14-3-3η with disease severity. Wilcoxon matched-pairs test was used to evaluate the significance of pre- to post-treatment changes. Mann–Whitney U test was performed for differences between treatment response groups. Fisher’s exact test was used to assess associations of 14-3-3η with a good response defined by European League Against Rheumatism criteria as well as remission defined by the Disease activity Score in 28 joints with erythrocyte sedimentation rate (DAS28-ESR) and the Clinical Disease Activity Index score.

**Results:**

14-3-3η-positive patients had more severe disease before the initiation of treatment. When combined with C-reactive protein (CRP), 14-3-3η positivity added significantly and incrementally to the identification of patients with high disease activity. 14-3-3η levels were significantly decreased at 1 year and were modifiable across all classes of therapeutics. Patients who reverted to negative 14-3-3η levels had better clinical response than patients who remained positive at 1 year or became positive. Pretreatment 14-3-3η levels informed 1-year DAS28-ESR remission in the TCZ-treated group, in contrast to the ADA, MTX, or TOF groups, while no differences in pretreatment 14-3-3η expression based on clinical response.

**Conclusions:**

14-3-3η is a modifiable marker in identifying patients with RA in a high disease state. Patients who achieve a negative 14-3-3η status following 1-year of treatment do better clinically with pretreatment 14-3-3η informing response to TCZ.

## Introduction

Because rheumatoid arthritis (RA) is a multifactorial disease with a heterogeneous presentation and disease course, identifying patients with aggressive early RA for prompt and appropriate treatment is critical to minimize irreversible joint destruction and disability [1]. If there is intolerance or an inadequate response to initial therapy with methotrexate (MTX) or another synthetic disease-modifying antirheumatic drug (sDMARD), treatment should be intensified to achieve remission or low disease activity. Numerous biologic therapies targeting different inflammatory pathways have been developed for the treatment of RA, including those that target tumor necrosis factor (infliximab, etanercept, adalimumab [ADA], certolizumab, and golimumab), T-cell costimulation (abatacept), B-cell depletion (rituximab), and the interleukin 6 receptor (tocilizumab [TCZ]). Recently, small-molecule kinase inhibitors have been developed as therapeutics for RA, including the Janus kinase inhibitor tofacitinib (TOF).

Importantly, even with early identification of RA and prompt initiation of treatment, up to 70 % of patients do not attain a satisfactory clinical response with therapy switches within and between drug classes. This heterogeneous response to therapy provides strong evidence that RA is likely caused by any one or a number of different biochemical pathways acting alone and/or in concert, resulting in the manifestation of common symptomology and disease presentation. Notably, the pathologic factors in early and late RA differ with respect to cytokine profiles [2, 3]. These differences, together with heterogeneous treatment response, indicate that there are fundamental differences in the pathological processes based on the stage of disease. This situation highlights the need for biomarkers that can assist with personalizing treatment strategies. Coupled with this information are the results of phase III clinical trials demonstrating that the pathways that drive the inflammatory process might be distinct from those that ultimately lead to erosive disease [4–8].

14-3-3 proteins are an evolutionarily conserved family of molecular chaperones that play a critical role in the regulation of intracellular functions, including proliferation, differentiation, and metabolism, among other functions. The 14-3-3 family consists of seven isoforms: alpha/beta (α/β), epsilon (ε), gamma (γ), eta (η), tau (τ), zeta (ζ), and sigma (σ). In 2007, Kilani et al. reported that, on the basis of immunoblot analyses, 14-3-3η was found in significantly higher amounts in the serum and synovial fluid of patients with inflammatory arthritis compared with healthy subjects [9]. In 2014, Maksymowych et al. reported that levels of 14-3-3η, which were quantified using a 14-3-3η enzyme-linked immunosorbent assay (ELISA), were detectable at significantly higher levels in patients with early and established RA than in healthy subjects and patients with various autoimmune disorders and other arthritides [10]. They also found, through receiver operating characteristic curve analysis, that levels ≥0.19 ng/ml were highly discriminative for RA, with increasing 14-3-3η positivity cutoffs providing greater discriminatory power for the identification of RA. They also reported that the 14-3-3η levels did not correlate with acute-phase reactants such as C-reactive protein (CRP) and that common factors present in the serum, including rheumatoid factor (RF), did not interfere with the quantification of 14-3-3η [10]. Other studies have further confirmed the differential expression and specificity of 14-3-3η for RA [11–14].

In the present study, we evaluated 14-3-3η serum expression levels in a cohort of Japanese patients with established RA before the initiation of therapy and following 1 year of treatment with commonly used therapies, each with a differing mechanism of action, including MTX, ADA, TCZ, and TOF, in daily clinical practice. The relationship between disease severity, outcome, and 14-3-3η expression was evaluated, along with the utility of14-3-3η as a marker in informing therapy response.

## Methods

### Patient cohort

Serum 14-3-3η was assessed in 149 Japanese patients with established RA, classified according to the American College of Rheumatology 1987 criteria [15] , before the initiation of therapy with MTX, ADA, TCZ, or TOF and following 1 year of treatment in daily clinical practice. The vast majority of patients in this study were women (86 %), and the cohort had a mean (standard deviation) age of 57 (15) years and a median (interquartile range [IQR]) disease duration of 51 (9–150) weeks. A total of 23 patients received MTX, 49 received ADA, 50 received TCZ, and 27 received TOF. Clinical examinations by a certified rheumatologist were completed for all patients before initiation of therapy and after 1 year of treatment. Assessments included Disease Activity Score in 28 joints with erythrocyte sedimentation rate (DAS28-ESR), Clinical Disease Activity Index (CDAI), Simple Disease Activity Index (SDAI), 28-joint tender joint count (TJC28), 28-joint swollen joint count (SJC28), Sharp/van der Heijde score (SHS), joint space narrowing (JSN), and erosion score. Serological assessments included erythrocyte sedimentation rate (ESR), CRP, RF, and anticitrullinated protein antibodies (ACPA). The study was performed in accordance with the Declaration of Helsinki. Written informed consent was obtained from all study participants, and ethical approval was received from the University of Occupational and Environmental Health, Japan.

### Serum 14-3-3η measurements

Serum 14-3-3η levels were measured using a quantitative 14-3-3η ELISA kit (Augurex Life Sciences Corporation, Vancouver, BC, Canada). A 14-3-3η cutoff of ≥0.19 ng/ml—the positivity cutoff established by Maksymowych et al. [10]—was used to define 14-3-3η positivity. Two additional 14-3-3η positivity cutoffs also described by Maksymowych et al. [10] were used: one at twice the positivity cutoff (≥0.40 ng/ml) and the other at four times the positivity cutoff (≥0.80 ng/ml).

### Statistical analyses

The Mann–Whitney *U* test was used to assess clinical and serological differences between treatment groups at initiation and after 1 year of therapy. The relationship of 14-3-3η to clinical measures was assessed using Spearman’s rank correlation coefficient. Contingency analysis provided the strength of association of 14-3-3η status with DAS28-ESR, CDAI, and SDAI categorization (i.e., remission or low, moderate, or high disease state). To assess the complementarity between 14-3-3η and CRP in identifying patients in a CDAI-defined high disease state, CRP positivity was defined as ≥10 mg/L. Patients were categorized as being either negative for both markers, positive for any one of the two markers, or positive for both markers. The Wilcoxon matched-pairs signed-rank test was used to evaluate the significance of pre- to posttreatment changes in 14-3-3η levels within therapy groups. An unpaired *t* test assuming equal variances was used to compare differences between groups with fewer than 10 patients. Fisher’s exact test was employed to determine the association between positivity of 14-3-3η and a good response as defined by European League Against Rheumatism (EULAR) criteria  [16] or remission as defined by DAS28-ESR. DAS28-ESR and CDAI remission were defined as scores <2.6 and ≤2.8, respectively. All statistical analyses were completed with Prism 6 (GraphPad Software, La Jolla, CA, USA) or JMP 11 (SAS Institute, Cary, NC, USA) software. A *p* value <0.05 denoted statistical significance.

## Results

### Positivity of 14-3-3η informs a worse disease state

Before the initiation of therapy, 110 patients (74 %) were 14-3-3η-positive based on the 14-3-3η positivity cutoff of ≥0.19 ng/ml (Table 1). 14-3-3η-positive patients had significantly higher median (IQR) DAS28-ESR, CDAI, SDAI, TJC28, and SJC28 scores at initiation than patients who were 14-3-3η-negative. 14-3-3η-positive patients also had significantly higher ESR, ACPA, and RF levels.

**Table 1:** Pretreatment patient characteristics

Variable	Entire cohort	14-3-3η-negative	14-3-3η-positive	*p* Value
Number of patients	149	39	110	
Categorical variables				
Sex (% female)	128 (86 %)	34 (87 %)	94 (85 %)	0.7937
RF status (% positive)	126 (85 %)	21 (54 %)	105(95 %)	<0.0001
ACPA status (% positive)	97 (65 %)	20 (67 %)^a^	78 (98 %)^b^	<0.0001
14-3-3η status (% positive)	110 (74 %)	0 (0 %)	110 (100 %)	<0.0001
Concomitant use of MTX (%)	117 (79 %)	34 (87 %)	83 (76 %)	0.1733
Continuous variables				
Age (yr)^c^	57.3 (14.7)	54.6 (16.1)	58.2 (14.1)	0.1924
RA duration (mo)	51 (9–150)	34 (9–132)	55 (11–159)	0.3791
MTX dose (mg/wk)	8.0 (5.0–10.0)	8.0 (6.0–10.0)	8.0 (1.5–10.0)	0.456
DAS28-ESR	5.35(4.40–6.45)	4.77 (4.10–5.86)	5.62 (4.64–6.57)	0.0101
HAQ^d^	1.07 (0.60–1.88)	1.00 (0.47–1.88)	1.13 (0.63–1.85)	0.7328
CDAI	22.2 (14.0–33.5)	16.0 (11.5–27.0)	24.7 (16.2–36.3)	0.015
SDAI	24.3 (14.3–37.4)	18.8 (11.7–32.7)	26.8 (16.7–38.6)	0.0238
TJC28	7.0 (4.0–13.5)	5.0 (2.0–12.0)	7.0 (4.0–14.0)	0.0327
SJC28	6.0 (2.0–9.0)	4.0 (2.0–9.0)	6.5 (2.8–10.0)	0.0466
SHS	26.5 (4.5–90.8)	14.0 (3.0–88.5)	31.3 (6.9–99.9)	0.1185
JSN score^e^	18.0 (3.0–45.5)	9.0 (1.5–42.5)	19.8 (4.1–47.4)^f^	0.11
Erosion score^e^	10.5 (2.0–45.5)	5.0 (2.0–40.5)	12.8 (2.5–53.0)^f^	0.108
ESR	44.0 (21.5–72.5)	35.0 (17.0–58.0)	47.5 (22.8–80.0)	0.0482
CRP (mg/dl)	0.83(0.23–3.13)	0.69 (0.20–2.70)	0.99 (0.26–3.44)	0.3769
RF	60.4 (27.4–171.8)	15.5 (5.8–43.2)	84.5 (41.9–242.1)	<0.0001
ACPA	100.0 (27.4–100)	16.9 (1.9–100)^a^	100.0 (52.0–100)^b^	0.0002
14-3-3η (ng/ml)	0.70 (0.17–5.96)	0.10 (0.04–0.13)	1.70 (0.49–11.0)	<0.0001

*Abbreviations: ACPA* anticyclic citrullinated antibodies, *CDAI* Clinical Disease Activity Index, *CRP* C-reactive protein, *DAS28-ESR* Disease Activity Score in 28 joints with erythrocyte sedimentation rate, *ESR* erythrocyte sedimentation rate, *HAQ* Health Assessment Questionnaire, *JSN* joint space narrowing, *MTX* methotrexate, *RF* rheumatoid factor, *RA* rheumatoid arthritis, *SDAI* Simple Disease Activity Index, *SHS* Sharp/van der Heijde score, *SJC28* 28-joint swollen joint count, *TJC28* 28-joint tender joint count

All *p* values were generated based on Mann–Whitney analysis, with the exception of age, which was generated using paired *t* tests.

^a^Data were available for 30 patients

^b^ACPA values were available for 80 patients

^c^Age is presented as the mean (standard deviation). All other variables are presented as the median (interquartile range).

^d^HAQ scores were available for 146 patients

^e^JSN and erosion scores were available for 147 patients

^f^JSN and erosion scores were available for 108 patients

**Fig. 1:**
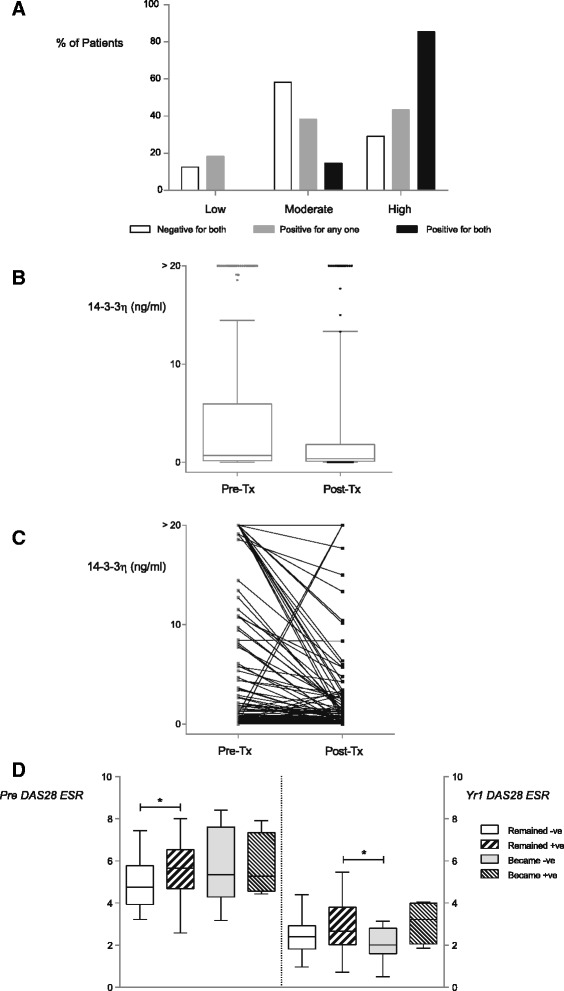
14-3-3η expression. **a** Clinical Disease Activity Index (CDAI)-defined disease states. The bar chart illustrates the relationship between 14-3-3η and/or C-reactive protein positivity and CDAI-defined disease status, **b** Changes between pre- and posttreatment. The box plot shows the change median levels of 14-3-3η over time, **c** Pairing by patient. The graph illustrates individual patients’ changes between pretreatment and posttreatment 14-3-3η serum levels. **d** Disease Activity Score in 28 joints with erythrocyte sedimentation rate (DAS28-ESR) based on changes in 14-3-3η expression. The box plot illustrates changes in median DAS28-ESR expression as 14-3-3η positivity changes over time. *Significance values were calculated using Dunn’s posttest

Spearman’s correlation analysis revealed that titers of 14-3-3η at therapy initiation correlated modestly with DAS28-ESR, CDAI, SDAI, JSN, TJC28, SJC28, CRP, and ESR values (Table 2). Contingency analysis revealed a strong and significant association at initiation between patients in higher 14-3-3η cutoff categories with moderate and/or high disease severity across the three different indices: DAS28-ESR, CDAI, and SDAI (Table 3). Because 14-3-3η had a modest correlation with CRP, the complementarity of these two markers in identifying patients with a CDAI-defined high disease status was assessed. As depicted in Table 3, using the ≥0.80 ng/ml cutpoint for positivity, 14-3-3η delivered a likelihood ratio (LR) of 14.0. Positivity for CRP based on ≥10 mg/L delivered a significant and strong LR of 20.5 (*p* < 0.0001). As illustrated in Fig. 1a and described herein, positivity for both 14-3-3η and CRP identified a significantly higher proportion of patients who are in a CDAI-defined high disease state than those who are positive for any one of the two markers or negative for both. The LR for CRP increased from 20.5 to 37.6 9*p* < 0.0001) when both markers were combined. This complementarity with CRP was also observed at the 0.19 ng/ml positivity cutoff for 14-3-3η, with the combined LR being 24.3 (*p* < 0.0001).

**Table 2:** Spearman’s correlation coefficients of pretreatment clinical variables with 14-3-3η

Variables	DAS28-ESR	SDAI	CDAI	HAQ	SHS	JSN score	Erosion score	TJC28	SJC28	14-3-3η	CRP	ESR
DAS28-ESR												
SDAI	0.91^a^											
CDAI	0.89^a^	0.99^a^										
HAQ	0.59^a^	0.54^a^	0.53^a^									
SHS	0.34^a^	0.28^b^	0.27^b^	0.43^a^								
JSN score	0.33^a^	0.28^b^	0.27^b^	0.41^a^	0.98^a^							
Erosion score	0.32^a^	0.24^b^	0.23^b^	0.43^a^	0.97^a^	0.91^a^						
TJC28	0.80^a^	0.86^a^	0.89^a^	0.45^a^	0.19^c^	0.19^c^	0.16					
SJC28	0.73^a^	0.84^a^	0.84^a^	0.33^a^	0.35^a^	0.33^a^	0.28^b^	0.69^a^				
14-3-3η	0.29^a^	0.24^b^	0.25^d^	0.05	0.16	0.18^c^	0.16	0.21^c^	0.26^b^			
CRP	0.58^a^	0.49^a^	0.36^a^	0.34^a^	0.32^a^	0.32^a^	0.32^a^	0.22^b^	0.29^a^	0.20^c^		
ESR	0.64^a^	0.37^a^	0.29^a^	0.34^a^	0.33^a^	0.32^a^	0.32^a^	0.18^c^	0.29^b^	0.27^b^	0.75 ^a^	

*Abbreviations: CDAI* Clinical Disease Activity Index, *CRP* C-reactive protein, *DAS28-ESR* Disease Activity Score in 28 joints with erythrocyte sedimentation rate, *ESR* erythrocyte sedimentation rate, *HAQ* Health Assessment Questionnaire, *JSN* joint space narrowing, *SDAI* Simple Disease Activity Index, *SHS* Sharp/van der Heijde score, *SJC28* 28-joint swollen joint count, *TJC28* 28-joint tender joint count

^a^ *p* < 0.0001

^b^ *p* < 0.01

^c^ *p* < 0.05

^d^ *p* < 0.001

**Table 3:** Pretreatment 14-3-3η positivity and association with disease state

		DAS28-ESR category^a^			SDAI category			CDAI category		
Cutpoint		≥0.19 ng/ml	≥0.40 ng/ml	≥0.80 ng/ml	≥0.19 ng/ml	≥0.40 ng/ml	≥0.80 ng/ml	≥0.19 ng/ml	≥0.40 ng/ml	≥0.80 ng/ml
Pretreatment	LR	10.6	16.3	25.2	3.7	9.7	16.3	6.2	8.8	14.0
	*p*-value	0.0011	<0.0001	<0.0001	0.16 (ns)	0.0077	0.0003	0.045	0.012	0.0009
1 yr	LR	10.1	5.1	2.6	2.2	1	1	2.2	1.1	0.8
	*p*-value	0.0064	0.079 (ns)	0.27 (ns)	0.34 (ns)	0.61 (ns)	0.61 (ns)	0.34 (ns)	0.57 (ns)	0.69 (ns)

*Abbreviations: DAS28-ESR* Disease Activity Score in 28 joints with erythrocyte sedimentation rate, *SDAI* Simple Disease Activity Index, *CDAI* Clinical Disease Activity Index, *LR* likelihood ratio, *ns* not significant

^a^Calculated based on 145 patients for pretreatment and 146 for 1 yr

### Levels of 14-3-3η are modifiable overtime

After 1 year of treatment, 97 patients (65 %) were 14-3-3η-positive. The data in Table 4 and Fig. 1b demonstrate that pretreatment 14-3-3η serum levels were significantly higher than 1-year levels across the whole group (*p* < 0.0001). As illustrated in Fig. 1c, the levels of 14-3-3η either increased, decreased, or stayed the same over time. Of the 110 patients who were positive for 14-3-3η before the initiation of treatment, 18 reverted or became negative (BN) based on the ≥0.19 ng/ml positivity cutoff, whereas 92 remained positive (RP). Of the 39 patients who were negative at treatment initiation, 5 converted at 1 year or became positive (BP) based on the 0.19 ng/ml positivity cutoff, whereas 34 remained negative (RN).

**Table 4:** 14-3-3η levels by therapy group at baseline and after 1 year of therapy

Group	Sample size	Pretreatment 14-3-3η	14-3-3η at 1 yr	*p* Value
Whole cohort	149	0.70 (0.17–5.96)	0.37 (0.11–1.82)	<0.0001
Adalimumab	49	0.62 (0.16–4.66)	0.38 (0.13–1.40)	<0.0001
Methotrexate	23	0.67 (0.17–1.89)	0.33 (0.11–1.44)	0.0101
Tocilizumab	50	0.43 (0.15–8.40)	0.27 (0.10–2.58)	0.0007
Tofacitinib	27	1.30 (0.21–9.46)	1.26 (0.11–3.07)	0.013

Median (interquartile range) 14-3-3η serum titers at baseline and at 1 yr are compared with the whole cohort and each therapy group. Corresponding Wilcoxon matched-pairs signed-rank test values were calculated

The Kruskal-Wallis test revealed a significant difference in DAS28-ESR at 1 year between the four 14-3-3η modifiability groups (stayed negative, BN, stayed positive [SP], BP) (*p* = 0.014). DAS28-ESR levels were not significantly different among the four different groups before the initiation of treatment. By post hoc Dunn’s testing, a significant difference (*p* ≤ 0.05) in median DAS28-ESR levels was demonstrated between BN patients (2.01; 1.60–2.80) and SP patients (5.27; 4.57–7.34) (Fig. 1d). The Mann–Whitney *U* test further highlighted this difference (*p* = 0.004). To assess whether a difference in 1-year DAS28-ESR existed between the BP group versus the BN group, an unpaired *t* test assuming equal variances revealed that mean DAS28-ESR 1-year levels were significantly lower in the 18 BN patients than in the 5 BP patients (2.09 ± 0.18 vs 3.09 ± 0.52; *p* = 0.033).

### Relationship of 14-3-3η to therapy

As shown in Table 4, the levels of 14-3-3η before the initiation of therapy and the posttreatment levels at 1 year were significantly different across all four classes of therapy. Response rates across the whole cohort based on DAS28-ESR-defined remission and a EULAR-defined good response were 55 % and 71 %, respectively. When we examined the differential expression of 14-3-3η by therapy response, 14-3-3η levels before the initiation of therapy emerged as significantly lower in the TCZ treated group that achieved DAS28-ESR remission. This finding was in contrast to those in the ADA, MTX, and TOF treated groups (Table 5), where no difference was seen in the levels of the 14-3-3η before the initiation of treatment. Interestingly, no significant differences in pretreatment CRP levels were observed across the whole cohort and within the different therapy groups. Similar to 14-3-3η, CRP levels at 1 year were significantly lower in the TCZ group that achieved a DAS28 ESR remission. For a EULAR-defined good response, whereas 14-3-3η baseline levels in these groups appeared different, the nonresponder subgroup was too small to return a significant difference.

**Table 5:** Clinical response after 1 year, by therapy

	All therapies		ADA		MTX		TCZ		TOF	
Patients (n)	149		49		23		50		27	
Pretreatment DAS28-ESR	5.35 (4.40–6.45)		5.33 (4.34–627)		4.23 (3.84–5.12)		5.43 (4.57–6.51)		6.34 (5.74–7.11)	
DAS28-defined remission	Y	N	Y	N	Y	N	Y	N	Y	N
Patients (n)	82	67	26	23	15	8	30	20	11	16
Pretreatment DAS28-ESR	5.00 (4.09–6.13)^a^	5.85 (4.90–6.77)	5.30 (4.06–5.83)	5.67 (4.82–6.77)	4.11 (3.75–4.43)	4.78 (4.24–5.27)	4.80 (4.23–6.27)^b^	6.02 (5.30–7.44)	6.60 (5.60–7.11)	6.28 (5.78–7.09)
Response rate	55 %		53 %		65 %		60 %		41 %	
Pretreatment 14-3-3η (ng/ml)	0.50 (0.16–4.95)	1.05 (0.19–7.75)	1.16 (0.20–9.06)	0.47 (0.13–2.14)	0.70 (0.17–1.89)	0.42 (0.16–14.55)	0.23 (0.09–3.03)^b^	4.02 (0.46–19.77)	1.05 (0.30–9.46)	1.56 (0.20–11.58)
1-yr 14-3-3η (ng/ml)	0.33 (0.10–1.45)	0.62 (0.14–3.11)	0.75 (0.14–2.96)	0.30 (0.13–1.17)	0.37 (0.11–0.62)	0.28 (0.07–18.33)	0.13 (0.08–0.75)^b^	1.52 (0.28–5.57)	1.24 (0.11–1.85)	1.27 (0.11–5.08)
Pretreatment CRP (mg/ml)	0.73 (0.15–3.12)	1.14 (0.26–3.40)	0.62 (0.14–5.01)	1.14 (0.40–5.32)	0.36 (0.06–0.83)	0.18 (0.08–1.34)	0.92 (0.14–2.18)	2.67 (0.40–3.50)	2.97 (0.56–5.22)	1.04 (0.33–2.98)
1-yr CRP (mg/ml)	0.03 (0.01–0.09)^b^	0.09 (0.02–0.37)	0.04 (0.02–0.13)^a^	0.14 (0.07–1.22)	0.05 (0.02–0.13)	0.12 (0.03–0.31)	0.01 (0.00–0.030)^c^	0.03 (0.01–0.18)	0.03 (0.02–0.16)	0.04 (0.03–0.28)
EULAR-defined good response	Y	N	Y	N	Y	N	Y	N	Y	N
Patients (n)	101	43	30	19	16	4	40	8	15	12
Pretreatment DAS28-ESR	5.32 (4.33–6.45)	5.77 (4.70–6.55)	5.33 (4.33–6.03)	5.32 (4.32–6.77)	4.21 (3.83–5.03)	4.42 (3.32–5.37)	5.02 (4.37–6.61)	5.75 (4.83–6.02)	6.60 (5.74–7.25)	6.10 (5.53–6.60)
Response rate	70 %		61 %		80 %		83 %		56 %	
Pretreatment 14-3-3η (ng/ml)	0.62 (0.16–5.02)	1.48 (0.20–12.72)	0.61 (0.12–3.61)	0.70 (0.24–5.84)	0.65 (0.12–1.03)	1.05 (0.20–14.82)	0.37 (0.16–7.33)	10.34 (0.19– > 20)	1.05 (0.30–8.16)	1.56 (0.20–14.02)
1-yr 14-3-3η (ng/ml)	0.28 (0.10–1.51)^b^	0.80 (0.27–5.99)	0.45 (0.11–1.45)	0.36 (0.22–1.41)	0.30 (0.10–0.47)^c^	7.39 (0.53– > 18.33)	0.18 (0.08–2.02)^b^	3.42 (0.39– > 20)	1.24 (0.11–1.85)	1.35 (0.19–9.06)
Pretreatment CRP (mg/ml)	0.96 (0.21 – 3.35)	0.64 (0.23 –3.08)	0.80 (0.27 – 5.33)	0.68 (0.30 – 4.20)	0.16 (0.05–0.80)	0.33 (0.10–1.34)	1.10 (0.20–3.11)	0.60 (0.16–2.99)	2.18 (0.77–4.28)	1.04 (0.26–3.58)
1-yr CRP (mg/ml)	0.03 (0.01 – 0.12)^c^	0.11 (0.03 – 0.50)	0.05 (0.02 – 0.14)^a^	0.13 (0.07 – 1.50)	0.04 (0.02–0.24)	0.13 (0.03–0.22)	0.01 (0.00–0.05)^a^	0.06 (0.02–1.36)	0.03 (0.02–0.16)	0.07 (0.02–0.28)

*Abbreviations: ADA* adalimumab, *CRP* C-reactive protein; *DAS28-ESR* Disease Activity Score in 28 joints with erythrocyte sedimentation rate, *EULAR* European League Against Rheumatism, *MTX* methotrexate, *TCZ* tocilizumab, *TOF* tofacitinib

Values shown are median (interquartile range)

^a^ *p* < 0.001

^b^ *p* < 0.01

^c^ *p* < 0.05

Fisher’s exact test revealed that pretreatment 14-3-3η at all positivity cutpoints was associated with the achievement of DAS28-ESR remission in TCZ-treated patients with pretreatment levels of ≤0.40 ng/ml, delivering the strongest LR of 15.67 (*p* = 0.0001). As DAS28-ESR levels at initiation were significantly lower in DAS28-ESR remitters in the TCZ group (Table 5), controlling for DAS28-ESR in a multivariable regression model demonstrated that a 14-3-3η level ≤0.40 ng/ml was an independent predictor of DAS28-ESR remission in TCZ-treated patients (LR = 10.24, *p* = 0.0014).

Because 14-3-3η and CRP levels were significantly lower at 1 year in DAS28-ESR remitters treated with TCZ, Fisher’s exact test was performed to assess the association of both of these markers with DAS28-ESR- and CDAI-defined remission. In both instances, positivity of 14-3-3η at 1 year, based on the cutpoint of 0.19 ng/ml, was associated with clinical remission (DAS28-ESR-defined remission LR = 6.96, *p* = 0.01; CDAI-defined remission LR = 7.14, *p* = 0.01), whereas CRP positivity defined as ≥10 mg/L was not.

## Discussion

In this cohort of Japanese patients with established RA, the results corroborate previously published findings that 14-3-3η-positive status corresponds with higher disease severity. We report, for the first time to our knowledge, that a positive status and higher levels of 14-3-3η based on different positivity cutpoints inform “moderate” and/or “high disease” activity states based on DAS28-ESR, CDAI, and SDAI scores. We also corroborate previously published findings that 14-3-3η has a modest correlation with the acute-phase reactants CRP and ESR [10, 17] and illustrate for the first time that the addition of 14-3-3η to CRP is significantly useful in identifying patients in a CDAI-defined high disease state. In this regard, the LR for moderate to high disease categorization based on CDAI increased from 20.5 to 37.6 when 14-3-3η was added to CRP. Although in this study we specifically evaluated 14-3-3η in RA, in other diseases it has been reported that elevated expression of other 14-3-3 isoforms is associated with more severe disease or worse outcomes, including but not limited to cancer and Creutzfeldt-Jakob disease [18–26].

Although higher expression of 14-3-3 proteins has been noted to be associated with less favorable outcomes across different pathologies, whether these proteins are causal or exist as a consequence of disease is yet to be fully elucidated. Of interest, Maksymowych and colleagues reported, on the basis of their in vitro and ex vivo experiments, that extracellular 14-3-3η, in a concentration-dependent manner using clinically relevant levels detectable in the serum of patients with RA, is capable of inducing both proinflammatory cytokines and chemokines and those factors that are directly involved in the degradation of cartilage and bone [17, 27]. More recently, they also reported that targeting 14-3-3η using an antibody-based approach in the collagen-induced arthritis mouse model delayed the onset of disease and reduced the overall disease severity [28].

In the present study, 14-3-3η levels at 1 year were also determined to be significantly lower across the whole cohort, as well as within each treatment group, when compared with the corresponding level before the initiation of treatment. Serial changes in 14-3-3η reflect the dynamic state of this marker wherein its modifiability over time, as well as its association with therapy response, is in clear contrast to RA biomarkers such as RF and/or ACPA, which are relatively undynamic with regard to treatment response [29]. We also report that reversion to a normal 14-3-3η state (i.e., ≤0.19 ng/ml) was associated with better clinical outcomes. In particular, patients who were 14-3-3η-positive at therapy initiation and reverted to a negative status at the 1-year follow-up had significantly lower DAS28-ESR scores than RP or BP patients. This association with disease activity is further corroborated by the fact that 88 % of 14-3-3η-negative patients at 1 year were in remission or in a low disease activity state compared with 66 % of 14-3-3η-positive patients. On the basis of these data, 14-3-3η may have utility as a marker of clinical response, and the impact of targeting a normal 14-3-3η level as part of a patient management strategy needs to be evaluated prospectively.

Because the ultimate goal for RA patient management is to achieve full clinical remission and halt radiographic damage and/or progression, more precise patient management through the use of markers such as 14-3-3η may assist in implementing treat-to-target strategies. Smolen et al. put forth a set of recommendations in which they stated that, with long-standing disease, as was the case for most patients in the present study, achieving low disease activity in those who are refractory to therapy is an important step in disease management, whereas in early disease, low disease activity should be considered a step toward clinical remission [30]. In the present study, we demonstrate that 14-3-3η levels at 1 year, across the whole cohort, were significantly lower in those who achieved a EULAR-defined good response. When evaluated in relation to the type of therapy administered, lower 14-3-3η pretreatment level was determined to be an independent predictor of DAS28-defined remission in patients who were treated with TCZ but not with any of the other classes of therapy. Moreover, 1-year 14-3-3η levels were associated with DAS28-ESR- and CDAI-defined remission. It is noteworthy that CRP levels at therapy initiation were not an independent predictor of DAS28-ESR-defined remission, nor were the levels at 1 year informative of DAS28-ESR- or CDAI-defined remission. On the basis of these findings, further studies in larger cohorts are warranted to examine the utility of 14-3-3η in predicting and monitoring response outcomes across different classes of therapy, more specifically in patients with early RA.

As this was an observational study, it has several limitations. First, the pretreatment characteristics of the MTX group were quite different from the other groups, with the patients being recruited in a consecutive manner. Second, the majority of patients in the ADA group and the TCZ group, as well as all patients in the TOF group, had histories of inadequate response to MTX. Thus, this reflects the shorter symptom duration and lower pretreatment DAS28-ESR in the MTX group. Last, it would have been ideal if the sample sizes and response rates across the different treatment groups had been balanced.

## Conclusions

Positivity of 14-3-3η is associated with more severe disease and combines with CRP to identify patients at higher risk. Serial decreases in 14-3-3η levels in response to therapy are associated with better clinical outcomes, whereas increases or sustained levels of the marker are associated with a worse prognosis. Pretreatment 14-3-3η level was an independent predictor of DAS28-ESR-defined remission in patients treated with TCZ.

## Abbreviations

ACPA: anticitrullinated protein antibodies
ADA: adalimumab
BN: became negative
BP: became positive
CDAI: Clinical Disease Activity Index
CRP: C-reactive protein
DAS28: Disease Activity Score in 28 joints
ELISA: enzyme-linked immunosorbent assay
ESR: erythrocyte sedimentation rate
EULAR: European League Against Rheumatism
HAQ: Health Assessment Questionnaire
IQR: interquartile range
JSN: joint space narrowing
LR: likelihood ratio
MTX: methotrexate
RA: rheumatoid arthritis
RF: rheumatoid factor
RP: remained positive
SD: standard deviation
SDAI: Simple Disease Activity Index
sDMARD: synthetic disease-modifying antirheumatic drug
SHS: Sharp/van der Heijde score
SJC28: 28-joint swollen joint count
SP: stayed positive
TCZ: tocilizumab
TJC28: 28-joint tender joint count
TOF: tofacitinib
